# 

**Published:** 1869-04

**Authors:** 


					﻿VOL. XXIII.
No. 4.
THE
Dental Register.
EDITED BY
J. TAFT & G. ATATT.
APRIL, 1869.
MONTHLY:
THREE DOLLARS PER ANNUM, IN ADVANCE.
CINCINNATI:
WRIGHTSON & CO., Printers, 167 WALNUT STREET.
JT. A TP AZ. TAFT. D.-ntifits, 117 IFV'.«r Fourth St.
				

